# Surface defect detection method for electronic panels based on attention mechanism and dual detection heads

**DOI:** 10.1371/journal.pone.0280363

**Published:** 2023-01-13

**Authors:** Le Wang, Xixia Huang, Zhangjing Zheng

**Affiliations:** Institute of Logistics Science and Engineering, Shanghai Maritime University, Shanghai, People’s Republic of China; National University of Computer and Emerging Sciences, PAKISTAN

## Abstract

Automatic detection of surface defects in electronic panels is receiving increasing attention in the quality control of products. The surface defect detection of electronic panels is different from other target detection scenarios and is a meaningful and challenging problem. Its main manifestation is that surface defects of electronic panels usually exhibit extreme irregularity and small target characteristics, which bring great difficulties to the task of surface defect target detection including feature extraction and so on. The traditional methods can only detect a very small number of defect classes under specific detection conditions. And due to the weak robustness of these methods, they cannot be applied in real production scenarios on a large scale. Based on this, this paper applies the target detection technique under deep learning to the surface defect detection scenario of electronic panels for the first time. At the same time, in order to make the designed target detection network applicable to the electronic panel surface defect detection scenario and to enhance the interpretability of the designed target detection network in terms of computer mechanism. We design a deformable convolution module with a convolutional self-attentive module to learn the offset and a dual detection head incorporating the SE (Squeeze-and-Excitation) mechanism for the irregular characteristics of electronic panel surface defects and the small target characteristics, respectively. Finally, we carried out a series of experiments on our own electronic panel defect data set, including comparison with the most advanced target detection algorithms and a series of ablation experiments against our proposed method. The final experimental results prove that our method not only has better interpretability, but also has better metric performance, in which the map_0.5 metric reaches 78.257%, which is an increase of 13.506 percentage points over YOLOV5 and 33.457 percentage points higher than Retinanet. The results prove the proposed method is effective.

## 1. Introduction

**Fig 1:**
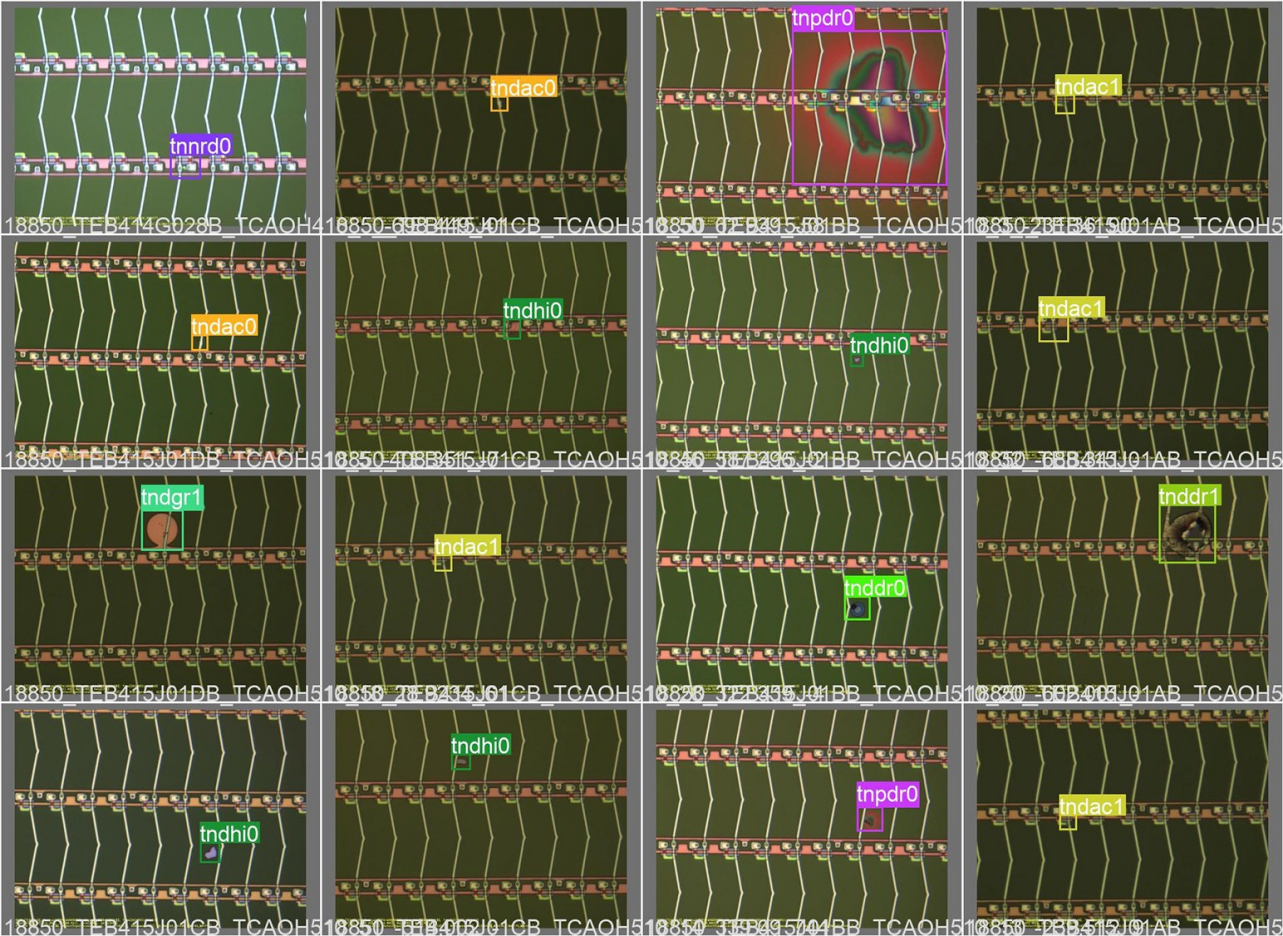
Common electronic panel surface defects.

In the production process of electronic panels, defects such as scratches, cracks, unevenness, heterogeneity, bright spots, and dark spots will inevitably appear in the production process because of the accuracy of the production equipment or the improper operation of the operator. If these defects cannot be detected in time, the quality of subsequent manufactured electronic products will be reduced, which will affect the customer experience and the market to a certain extent. Therefore, how to accurately detect the defects on the surface of electronic panels is a problem well worth studying. The common surface defects of electronic panels are shown in Fig 1. In all electronic panel pictures, the picture size occupies 1392*1040 pixels, while the pixel size occupied by various types of surface defects is randomly distributed between 0*0 and 1392*1040. The traditional manual testing methods depend greatly on inspectors’ quality, and some surface nondestructive tests have high requirements for inspection equipment and are susceptible to the influence of the accuracy of the testing equipment and the interference of the testing environment. However, these methods have the disadvantages of high costs, low accuracy and low efficiency. After developing to the machine vision detection stage, the problems of detection accuracy, detection efficiency, and detection cost have been solved to a certain extent. However, traditional machine vision methods rely too much on the experience of algorithm designers in the feature extraction process, making the network model designed to detect surface defects in electronic panels insufficient generalization capability, and often shows poor detection results when applied to surface defects of different backgrounds, and still needs to be improved in terms of detection performance.

In 2006, Hinton et al. [1] proposed the deep learning method, which promoted the development of various fields including image processing. The two-stage series of detection methods proposed by [2–5] and the one-stage series of detection methods proposed by [6–9] have also appeared in the field of target detection. These two-stage and one-stage target detection networks are supervised networks, in addition to the unsupervised and self-supervised target detection networks proposed by [10–13] et al. These detection methods have achieved excellent results in map, recall, frame rate and other indicators respectively. But these methods are specially designed for natural scenes data sets, such as MS COCO data sets [14] and PASCAL VOC data sets [15]. These scenes have rich data sets and high quality of images. The network can be fully trained easily and it is easy to extract features from the detection targets in the data. However, when these methods are applied to the surface defect detection of electronic panels, they usually have poor detection results. Through our analysis, we found that there are three main reasons for this difference. First, the sample of electronic panels with surface defects is small, it is difficult to collect a large-scale dataset of electronic panel surface defects. And small sample datasets are difficult to achieve better results even when placed on a very superior network. Second, some of the electronic panel surface defects are small, resulting in small differences between the defects and the background, the available features are limited, and their semantic information appears in a shallower feature map, and as the network deepens, their detailed information may disappear completely, which can be detrimental to the task of detecting defective targets on the surface of electronic panels, especially in the localization task with extremely poor results. Third, almost all classes of electronic panel surface defects exhibit extreme irregularities, and the shapes of defects of the same class are randomly distributed on the surface of electronic panels, and it is difficult for the traditional networks composed of standard convolution to effectively extract the features of these defects.

Recent improvements considered to solve the above three problems, [16,17] proposed an anomaly detection approach that requires only normal samples in the training process, reducing the dependence on the number of defective samples, but does not give further solutions to the defect irregularity and small target problems. The segmentation-based method proposed by [18,19] solves the problem of defect irregularity to a certain extent, but its model is too complex and the effect is still not satisfactory in scenarios with more small targets and defect types. To address these issues, we conducted the development of a surface defect dataset for electronic panels and designed a supervised deep learning network applied to surface defect detection of electronic panels, which features a better explanation of how deep learning networks work in surface defect detection of electronic panels while ensuring detection results. This supervised network consists of three parts: Backbone, neck, and Double T-head. In particular, the backbone stage consists of a conventional standard convolution module and and our specially designed T-deformable convolution module, which solves the problem of difficult feature extraction in electronic panel surface defect detection due to defect irregularities. Meanwhile, at the end of the network, a Double T-head incorporating the SE [20] mechanism (Squeeze-and-Excitation) is designed to solve the problem of difficult detection of small targets.

In summary, our contributions mainly include the following aspects:

1. In order to solve the problem of small sample size in electronic panel surface defect detection, we developed an electronic panel surface defect dataset. The images in our dataset come from the real environment of the factory, with a total of 6155 images and 64 defect categories, which are labeled using labelImg annotation software.
2. In order to solve the problem of irregular defects in electronic panel surface defect detection, we design the T-deformable convolution module in the backbone stage of the network to effectively extract the features of irregular surface defects, so as to improve the detection of electronic panel surface defects.
3. In order to solve the problem of difficult detection of small targets in electronic panel surface defect detection, we designed Double T-head at the end of the network to make up for the shortage of single detection head in detecting small targets and improve the detection effect under small target defects.

The structure of this paper can be divided into the following four parts:The first part explains the problems of existing target detection networks that cannot be applied in the detection of surface defects in electronic panels, the advantages and disadvantages of existing surface defect detection and our contributions. In the second part, we outline the related work, which mainly includes supervised defect detection methods, unsupervised defect detection methods, semi-supervised defect detection methods and segmentation-based methods. In Section 3, the structure of the supervised network we have designed is described in detail. It should be noted that Sections 3.2 and 3.3 introduce the structure of the T-deformable convolution module and the Double T-head module, respectively, and the rest of Section 3 presents other technical details included in the network. In Section 4, we demonstrate the advantages of the proposed approach through comparative and ablation experiments as well as theoretical analysis.

## 2. Related work

In this section, related methods of surface defect detection will be introduced, including supervised and unsupervised surface defect detection methods, and semi-supervised surface defect detection methods will also be introduced.

### 2.1 Supervised defect detection method

Most of the supervised defect detection methods are mainly improved on common target detection models developed for common data sets [21–24]. Among them, it is worth mentioning that the end-to-end method for steel plate defect detection proposed by He et al. [21] adopts feature fusion strategy to improve the richness of the extracted features by fusing multi-level feature information of the defect target, but fails to solve problems such as small samples and small targets in the field of surface defect detection; Wang et al. [22] proposed a joint detection CNN architecture consisting of a global frame and a sub-frame frame for fabric defect detection, whose detection performance has been greatly improved compared with the traditional algorithm, but it is still not optimal; Hu et al [25] applied the Faster-RCNN, a target detection network in a generic scenario, to PCB surface defect detection, only replacing the backbone network and modifying the prediction anchor, but there were no other innovative improvements, so the detection results were not satisfactory; Qiu et al. [23] adopted the full convolution (FCN) method to predict the defect area at the pixel-level, and used depth-wise, Pointwise convolutional layer, strided depth-wise convolutional layer and up-sampled depth-wise convolutional layer instead of standard convolutional layer, pooling layer, and deconvolutional layer. This method has greatly improved the detection accuracy and detection performance, but because the proposed algorithm is based on local information, its detection ability on structural defects is weaker than that on texture defects which needs to be improved.

### 2.2 Unsupervised defect detection methods

In recent years, in order to solve the problem of insufficient defect samples in surface defect detection and the difficulty of defect feature extraction, some unsupervised methods have been gradually proposed [26–30]. For example, Mei et al. [26] proposed a method of detecting and locating defects only after training with non-defective samples to detect fabric defects, which is mainly reconstructed by a network with a convolutional noise reduction autoencoder. This method effectively solved the problem of small samples, but the robustness of its model is poor. When the same type of defects shows different shapes, sometimes the defect category cannot be accurately detected; Zhao et al. [31] proposed an unsupervised method for fabric defect detection. This method constructs a reconstruction model through an autoencoder and GAN network, and then the defect image was input into the model to obtain a defect-free image. Then compare the restored non-defective image with the original image pixel by pixel to obtain the defect location. It only needs normal samples for training, and has a good application prospect in the project landing. However, due to the inevitable introduction of noise in the network reconstruction, detection accuracy and detection speed still need to be improved; Kim et al [32] proposed an unsupervised PCB surface defect detection network based on a jump-connected convolutional autoencoder. Their deep autoencoder model was trained to decode raw non-defective images from defective images, and then compare the decoded images with the input images to determine the defect locations. Its localization of defects is good, but it does not perform the classification work of defects.

### 2.3 Semi-supervised defect detection method

Although the unsupervised defect detection method solves the problem of insufficient defect samples, it also sacrifices the detection accuracy and detection rate to a certain extent, and the generalization ability of the model is insufficient, so the semi-supervised defect detection method is proposed [28,33–35]. For example, Di et al. [28] proposed a semi-supervised learning method combining convolutional autoencoder (CAE) and semi-supervised generative adversaria adjunctive network to classify defective samples. Compared with traditional methods, the CAE-SGAN method can make full use of sample images of the steel surface (labeled and unlabeled images), which improves the defect classification accuracy under limited training samples. However, the performance of this method is not optimal, and there are many deficiencies in defect location. Gao et al. [33] proposed an improved semi-supervised learning method with pseudo-labels using convolutional neural networks (CNN) to identify steel surface defects, which achieved 86.72% map in the dataset of a steel company, but its robustness was poor and the performance on different datasets varies greatly. Although semi-supervised learning has solved some problems in object detection and has some application research, there is still a lot of room for improvement in surface defect detection of electronic panels.

### 2.4 Segmentation-based defect detection method

Unlike the detection-based approach, the segmentation-based surface defect detection method converts the surface defect detection task into a semantic segmentation or even an instance segmentation problem between defective and normal regions, which can not only finely segment the defective regions, but also obtain the location, category, and corresponding geometric properties (including length, width, area, profile, center, etc.) of the defects, but this also puts higher demands on the network structure. This also imposes higher requirements on the network structure. The more classical segmentation-based methods are the semantic segmentation method based on FCN [36] and the instance segmentation method based on Mask R-CNN [37]. In recent studies, segmentation-based methods have been applied to various surface defect detection scenarios [18,19,38–41], for example, Gao et al [39] used an attention-based and multi-branch encoder-decoder structure and finally achieved pixel-level surface defect by fusing adjacent feature maps of each layer and integrating the highest layer segmentation, but its encoding and decoding process caused partial information loss, and only 73.7%, 85%, and 60.1% accuracy on the three datasets of MT, RSDD, and CFD.Su et al [40] used normalized flow in the segmentation process to determine whether defects exist on the product surface, and used multi-scale feature fusion alignment to initially locate the defects, then combined with gradient and maximum Li et al [41] proposed an effective backbone structure (Res2Net-Mish-BN-101) and designed an enhanced BiFPN, and applied a GAN-based defogging model to image pre-processing. The final application is for defect detection of sewer pipes, which achieves a 7.3% improvement in MAP compared to the original network, but the model is too complex and needs a lot of improvement work to achieve practical application. Ling et al [18] improved the effectiveness of some small target defects in PCB surface defects by using a segmentation model consisting of two encoders and decoders sharing weighted values, which better recovered the spatial information on multiple output layers, but did not give a better solution for the detection of irregular defects. In general, these methods provide us with good research ideas by combining some of the latest target detection research results, but there is still room for improvement in the number of defect detection categories, especially for small target areas such as electronic panel surface defects.

## 3. Proposed method

In this section, a better understandable target detection network structure for scenarios such as extreme and small target variations of electronic panel surface defects will be presented, specifically the T-deformable convolution module and Double T-head we designed, as well as the loss function and some training techniques in the network will be described in detail.

### 3.1 Network structure

**Fig 2:**
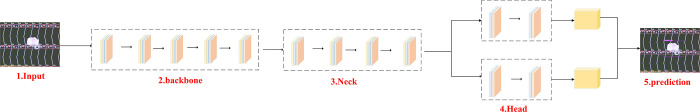
A kind of supervised target detector applied to the detection of surface defects in electronic panels. It includes Backbone, Neck, and Double T-head with features extracted by T-deformable convolution module, which will be described in detail in Chapter 3.

**Fig 3:**
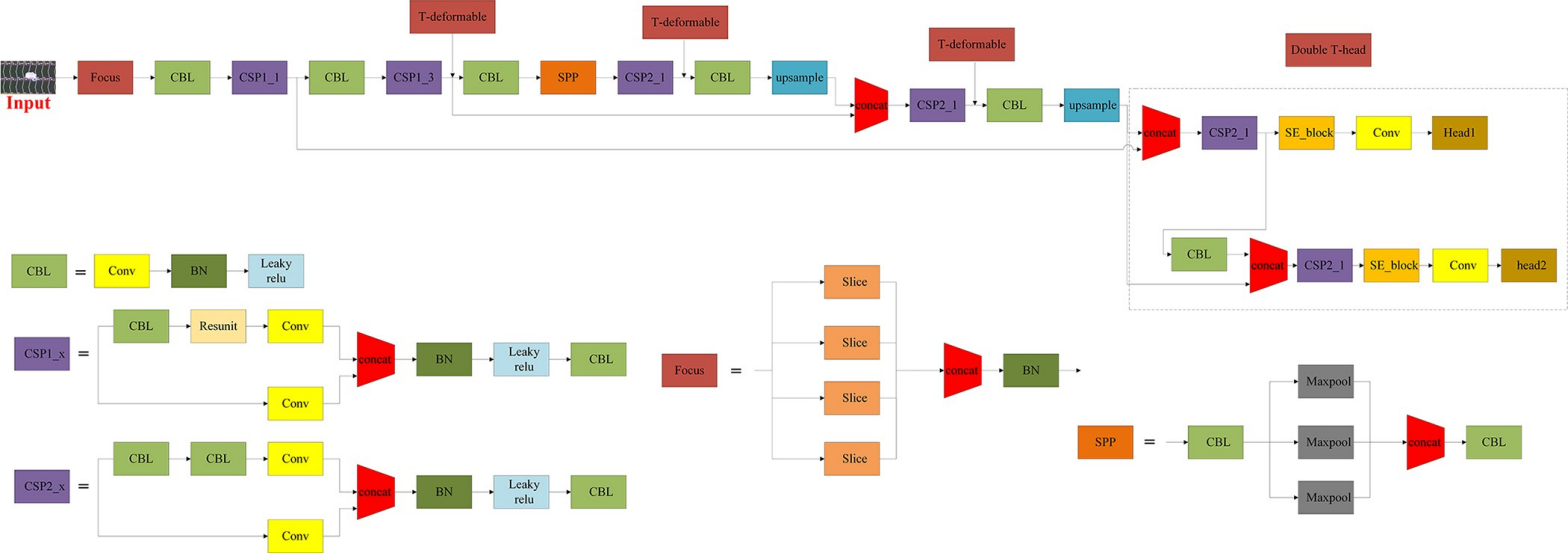
A more detailed network structure.

As shown in Fig 2, considering the high requirements for detection speed in practical application scenarios of electronic panel surface defect detection, our network structure uses many components of the one-stage framework. And on this basis, the applicability of the network in irregular target and small target scenarios is considered and better understandability is sought. Therefore, the T-deformable convolution module for feature extraction and Double T-head were redesigned, which is mainly designed for the problem of extremely irregular and small target surface defects of electronic panels, and the real time of practical deployment in subsequent projects. In general, the network structure consists of Backbone, Neck and Head. Backbone adopts Focus and CSP, in which the T-deformable convolution module designed for the characteristics of extreme irregularity of electronic panel surface defects is embedded, so that the network has strong robustness in extracting the characteristics of extreme irregularity of electronic panel surface defects. Neck adopts FPN and PAN to better utilize the feature information extracted by Backbone. Head adopts our specially designed dual detection head structure, which has improved the effect of detection in all types of example targets. A more detailed network structure is shown in Fig 3. It is worth mentioning that we use this network structure in the subsequent training and testing process.

### 3.2 T-deformable convolution module

**Fig 4:**
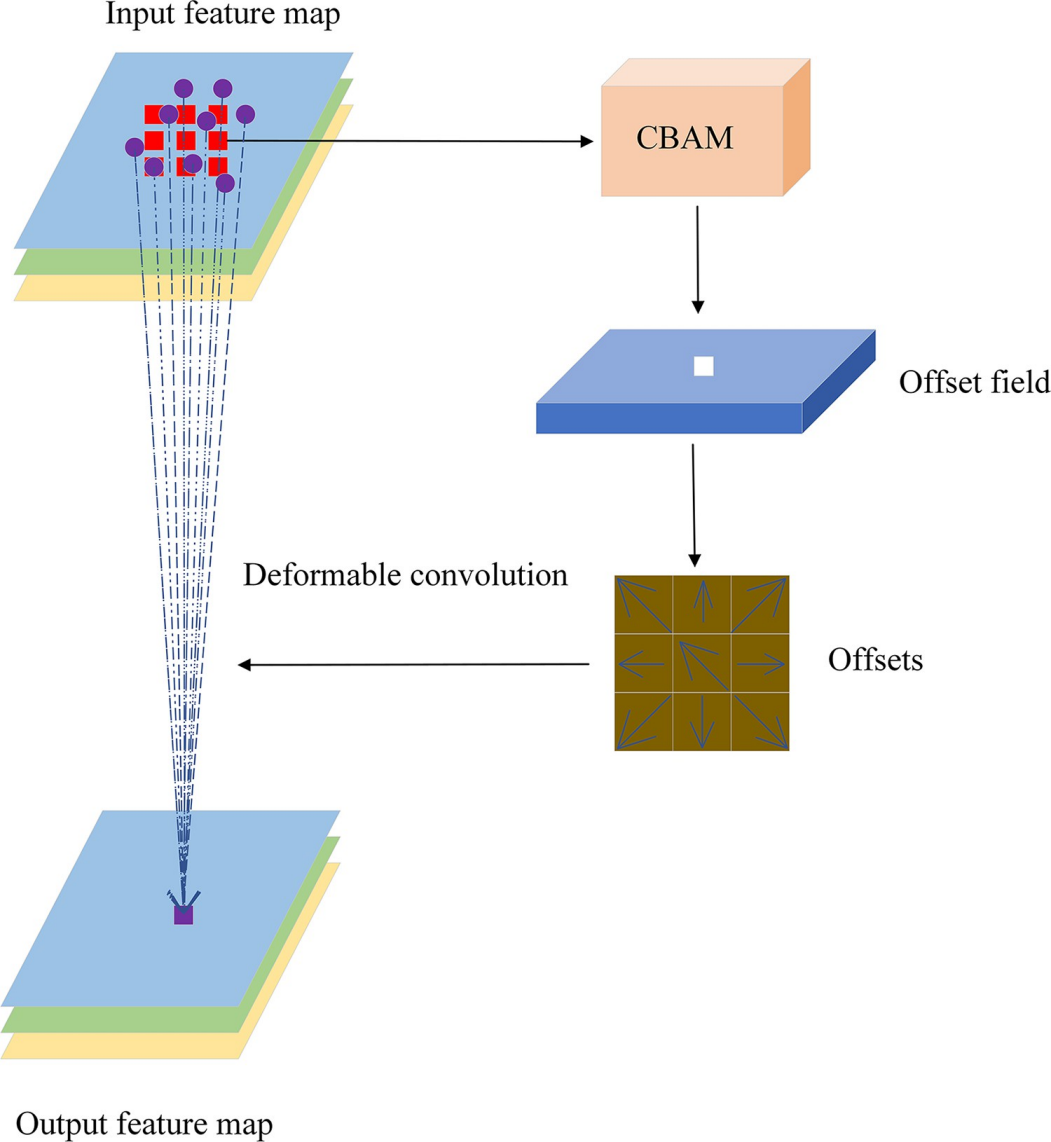
The deformable convolution module of the fusion convolution self-attention module. The left side is the feature extraction branch, and the right side is the offset learning branch. The offset is learned by the CBAM module.

In order to make our target detection network suitable for the detection scenario of irregular electronic panel surface defects and to give the network better interpretation performance, we have rethought the feature extraction mechanism of the defect detection network and designed the T-deformable convolution module as shown in Fig 4, which enables the network to accurately extract the features of irregular defect targets. As shown in Fig 3, we have used three T-deformable Convolution modules in the network, inserted behind the first CSP1_3 module, the first and the second CSP2_1 modules, respectively. In the T-Deformable Convolution module, the convolutional self-attention module learns the offset of the sampling point of the convolution kernel in the input feature map, and then sends the learned offset to the branch of feature extraction to accurately extract the deformed features. The traditional standard convolution process can be viewed as using convolution module sampling to model the feature information of the target in the image, and its mathematical principle can be expressed as:

$$y(p_{0})=\sum_{p_{n}\in R}w(p_{n})∙x(p_{0}+p_{n})$$ (1)

**Fig 5:**
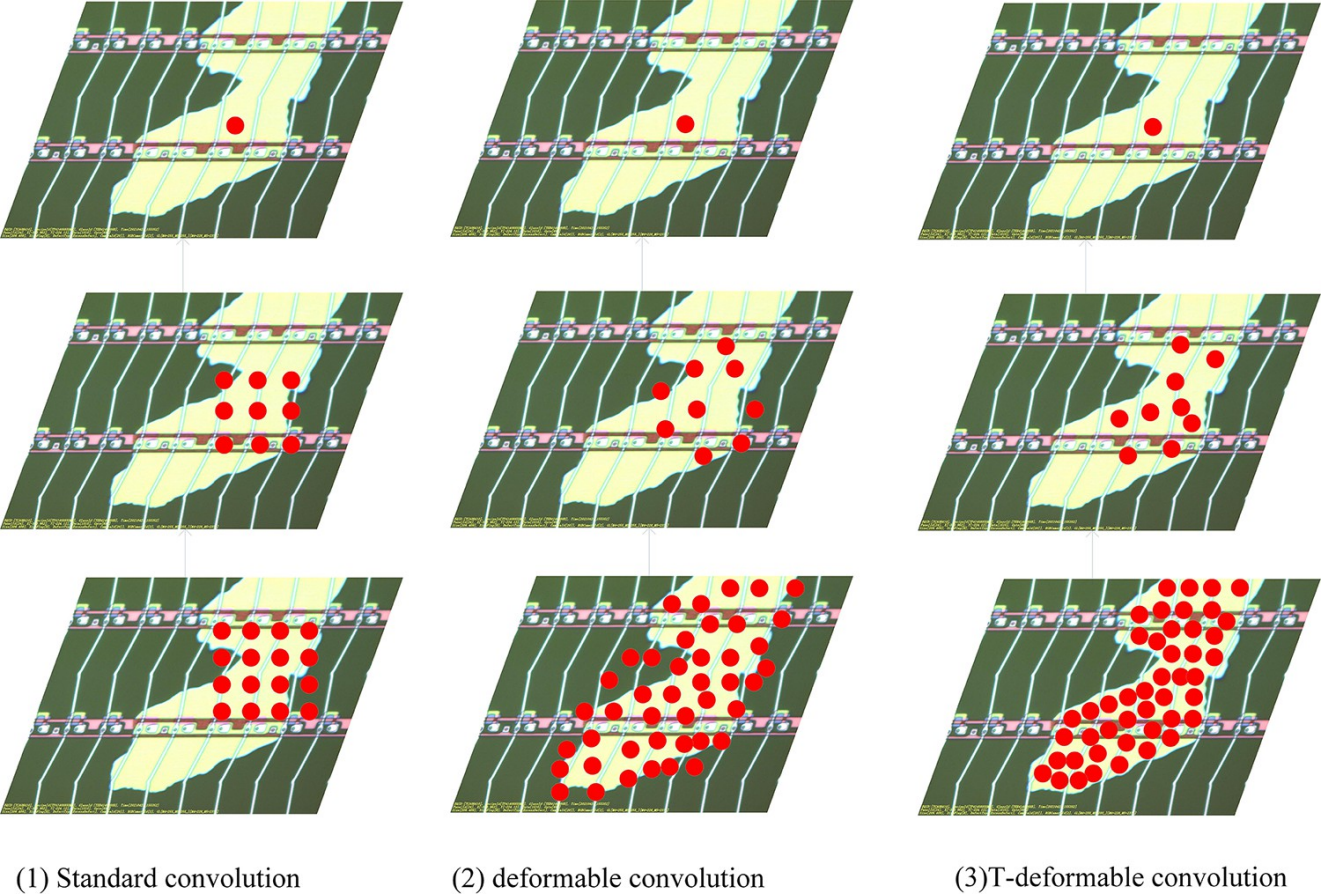
The difference between Standard convolution, deformable convolution, and T-deformable convolution. The left, middle, and right sides represent standard convolution, deformable convolution, and T-deformable convolution respectively. The upper, middle, and lower sides represent the activation unit on the feature map, 3 ×3 filter, sampling position.

For each position *p*_0_ on the output feature map, the feature information it represents is enumerated by the perceptual field size and its dilation factor R to enumerate the sampled information of the picture within its convolution size. This results in its sampled information being fixed and cannot effectively model irregular defects, whose sampling process is shown in (1) in Fig 5. The solution proposed by Dai et al [42] is to add an offset Δ*p*_*n*_ to the sampling process so that the sampling process becomes:

$$y(p_{0})=\sum_{p_{n}\in R}w(p_{n})∙x(p_{0}+p_{n}+\Delta p_{n})$$ (2)

Its better solves the feature extraction problem of irregular targets, but its feature extraction branch and the offset learning branch of sampling points are composed of standard convolution, but the standard convolution in learning the offset Δ*p*_*n*_, due to its limited spatial modeling ability of irregular defects, leads to its insufficient deformation ability or deformation ability far beyond the region of interest, which will cause the learning of the offset is not accurate enough. thus making the feature extraction information missing or affected by irrelevant image contents, whose sampling process is shown in (2) in Fig 5. For this reason, we propose the T-deformable convolution module, and use the CBAM module to perform the learning of the offset Δ*p*_*n*_. In our T-deformable module, the CBAM attention module is composed of channel attention module and spatial attention module. The channel attention module generates a channel attention map by exploiting the inter-channel relationship of features, and due to the each channel of feature map is considered as a feature detector, the channel attention focuses on the content of the given input image and is used to determine whether the location is defect or background. This spatial attention module focuses on the part information of position, which is supplementing the channel attention, which encodes the emphasized or suppressed position. The two attention modules of channel and spatial calculate complementary attention, pay attention to the content information and the position information of the target respectively, and accurately judge whether the current position and the surrounding position of the current position are defects or backgrounds. Thus, the offset of the sampling points of the input feature map is accurately determined, and the features of irregular defects are accurately sampled according to the different shapes they exhibit to avoid the influence of irrelevant contents. And to solve the phenomenon of false detection and missed detection that often occurs when the target detection network detects irregular defects on the surface of electronic panels. The structure of the T-deformable Convolution module is shown in Fig 4, and its feature extraction process is shown in (3) in Fig 5.

### 3.3 Double T-head

**Fig 6:**
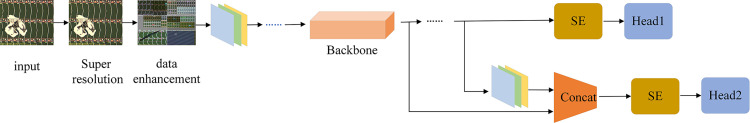
Double T-head structure, in addition to head1, head2 is also designed to enhance the detection of small target defects.

In order to make our target detection network suitable for small target detection scenarios in electronic panel surface defects, the Double T-head shown in Fig 6 is designed. In the Double T-head module, considering that some defect targets are relatively small among the surface defects of electronic panels, Head2 is additionally designed to address the deficiencies of the network in detecting small target defects, in addition to the conventional detection head1. Specifically, Head1 consists of a Concat operation, CSP2_1 module, SE block, and convolution block in order. Head2 has two inputs, one input is the output of the head1 branch through the CSP2_1 module and then through the CBL module, and the other input is the output of the third deformable convolution module through the CBL module and the upsampling module in turn. Both inputs go through the Concat operation in turn and then through the CSP2_1 module, the most important SE block, the convolution block, and finally to the second detection head. In particular, it is important to mention that the SE mechanism module in front of these two detection heads is the most important part of the entire dual detection head. The SE mechanism is divided into two steps: squeeze and excitation. The first step is squeeze, i.e., the global compressed feature amount of the current feature map is obtained by performing a global average pooling operation on the convolved feature map. The second step is excitation, where the weights of each channel in the mapped features are obtained through a two-layer fully connected bottleneck layer structure, and finally the superimposed weighted feature map is used as the input to the next layer. Because the same feature channel does not have the same importance for discriminating whether a feature belongs to the defective target or the background, we enable the detection head to focus more accurately on whether the input feature information is a defective target or a background by inserting an SE module that gives the learnable weights corresponding to all feature channels of the mapped features to distinguish the importance of the defective target and other background targets. With the weights of importance of feature channel, the actual true feature map to be obtained is equivalent to the weights of importance of feature channel multiplied by the original values of the features on each channel.

**Fig 7:**
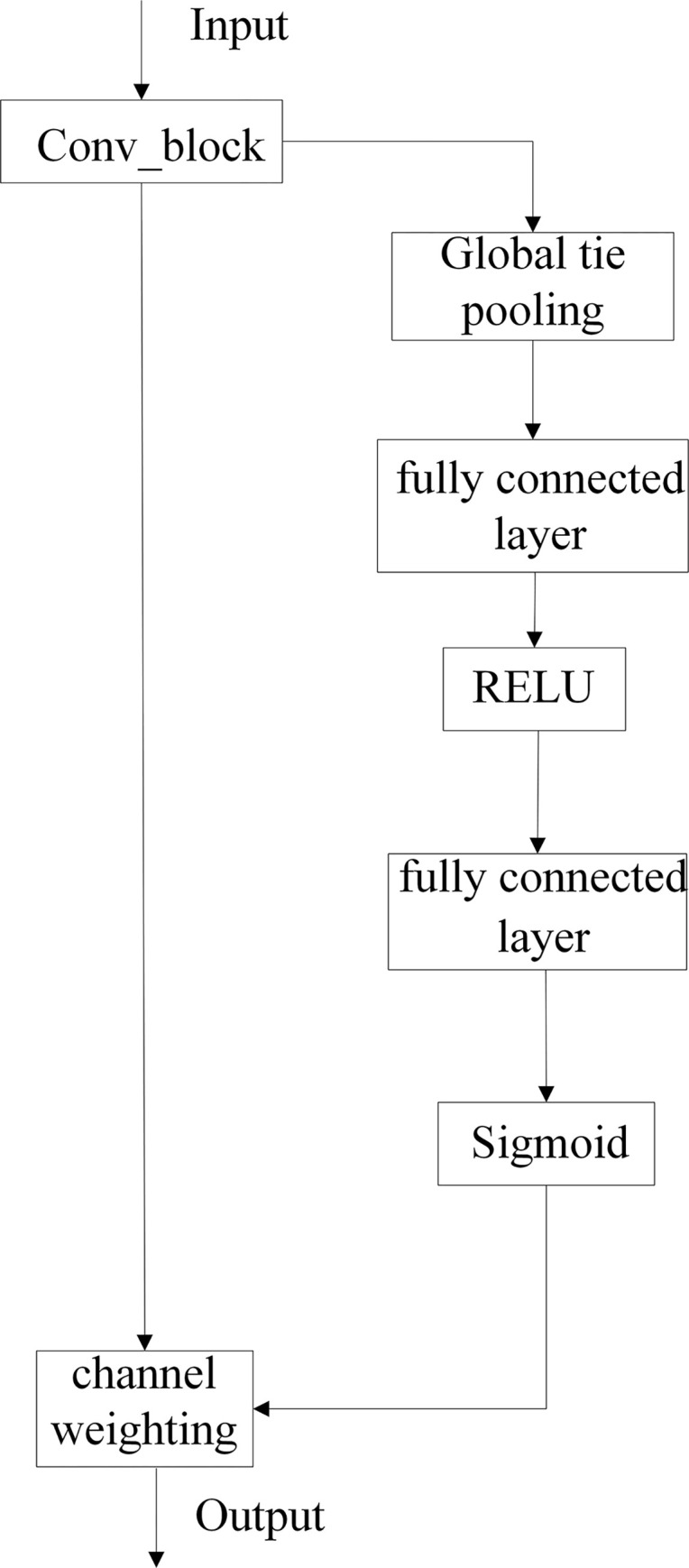
SE module structure.

The network structure of the SE module is shown in Fig 7. The input feature maps are convolved by the convolution module and then subjected to a global average pooling operation to obtain a sequence of real numbers of length M. So that the feature maps on each channel have a global sensory field. The shallow feature maps with smaller global sensory fields can use the global information to improve the feature extraction capability of the network and obtain richer semantic information of the images. Then a real sequence of length M is input to the fully connected layer, which reduces the dimension of the sequence to $\frac{1}{r}$. Then the number of channels is increased by using the ReLU activation function, and the weight coefficients of the channels are calculated by using the Sigmoid activation function. Finally, the weight coefficients are multiplied by the corresponding feature channels to update the feature map. This operation can enhance the receptive field at the detection side, increase the weights of the feature channels related to the target to be detected, effectively suppress the feature channels that are not related to the target to be detected, improve the semantic information of the feature map, and then improve the detection accuracy of the defective target.

In general, head1 can adapt to most of the defect detection tasks, head2 is designed with more consideration to head1’s susceptibility to false detection and missed detection when detecting small-scale defect targets. Therefore, the following detection head splices more layers of information, and the information obtained is richer. The SE mechanism can increase the receptive field of the feature map, which also helps head2 to obtain more feature information and improve the accuracy of electronic panel surface defect detection. At the same time, the channel attention module in the SE mechanism can feed the feature information of small targets into the second detection head precisely. And in order to distinguish the two detection heads, the stride of the first detection head and the second detection head are set to 76×76 and 38×38, respectively. With the setting of these modules, relying on dual detection heads can greatly improve the detection of target defects in electronic panel surface defects accuracy.

### 3.4 Loss function

It is well known that the target detection task contains a classification subtask and to a regression subtask, which aim to distinguish the category and to calibrate the location of the target, respectively, and the training process is usually optimized with classification loss and regression loss at the detection end. In the supervised electronic panel surface defect detection network we designed, three loss functions need to be used, and we refer to the target detection loss functions proposed in the literature [9], which will be described in detail in this section, and they are classification loss, localization loss, confidence loss.

#### 3.4.1 Classification loss

In the target detection task, the classification loss function is used to guide the learning of the classification task, and since we have a large variety of data and it is difficult to quantify the relationship between different categories, we only focus on the probability of whether a single category is correctly predicted, and the classification loss function used can be expressed as:

$$L_{1}=-\frac{1}{n}\sum \left(y_{n}\times lnx_{n}+(1-y_{n})\times \ln\left(1-x_{n}\right)\right)$$ (3)

Where n represents the total number of categories, *x*_*n*_ is the predicted value of the current category, and *y*_*n*_ is the label 0 or 1. Because the dataset selected in this experiment contains 64 types of defect samples, n takes 64. And the operation of sigmoid is required before the incoming value *x*_*n*_, the sigmoid formula is as follows:

$$S(x)=\frac{1}{1+e^{-x}}$$ (4)

#### 3.4.2 Localization loss

In the object detection tasks, localization loss is mainly used to guide the learning of regression sub-task. At present, the most popular loss functions for regression of the bounding box in the object detection task are IOU [43], GIOU [44], and DIOU [45]. Their mathematical expressions are as follows Formulas (5)、(6)、(7) are shown.


$$IOU=\frac{\left|B\cap B^{gt}\right|}{\left|B\cup B^{gt}\right|}$$ (5)

Among them, B and *B*^*gt*^ represent the prediction frame and the real frame, respectively.


$$GIOU=IOU-\frac{\left|C(B\cup B^{gt})\right|}{\left|C\right|}$$ (6)

Where C represents the smallest rectangular box containing all B and *B*^*gt*^.


$$DIOU=IOU-\frac{\rho^{2}(B,B^{gt})}{d^{2}}$$ (7)

Where *ρ*^2^(*B*, *B*^*gt*^) represents the Euclidean distance between the center point of the predicted frame and the real frame, and *d*^2^ represents the distance between the upper left corner and the lower right corner or the lower left corner and the upper right corner between the predicted frame and the real frame.

According to the regression triad of target detection, overlap area, centroid distance, and aspect ratio, the above loss functions either only consider the overlap area or the distance between the predicted frame and the centroid of the real frame, and considering only one of these factors is often not enough, which will lead to less accurate detection of the trained model on the test set, therefore, we use a more stringent constraint, and reference [46] additionally adds an aspect ratio constraint. The localization loss is replaced with:

$$L_{2}=1-IOU+(\frac{\rho^{2}(B,B^{gt})}{d^{2}}+\alpha *\nu )$$ (8)

$$Where\;\nu =\frac{4}{\pi^{2}}{\left(tanh\frac{w^{gt}}{h^{gt}}-tanh\frac{w}{h}\right)}^{2}$$ (9)

$$\alpha =\frac{v}{(1-IOU)+v}$$ (10)

*w*^*gt*^ and *h*^*gt*^ represents the length and width of the real box, respectively, w and h represent the length and width of the predicted box, respectively.

#### 3.4.3 Confidence loss

In order to measure the confidence of predicting a certain category in the classification results, confidence loss is also added, which is expressed as ***L***_**3**_, and ***L***_**3**_ is consistent with ***L***_**1**_. But ***L***_**3**_ internally encapsulates the Sigmoid operation inside, and no separate Sigmoid operation is required.

#### 3.4.4 Joint loss

Among the three above three loss functions, the contribution of the learning ability of different loss functions to the network may be inconsistent. In order to solve this problem, we use joint loss to train the entire network. The joint loss is expressed as follows:

$$L=\beta L_{1}+\gamma L_{2}+\delta L_{3}$$ (11)

Among them, β, γ, and δ are hyperparameters representing the importance of the three loss functions of *L*_1_, *L*_2_, and *L*_3_ to the entire loss, which can be adjusted during training.

### 3.5 Other metrics

#### 3.5.1 Image pre-processing

The characteristics of electronic component images are very different from those of public datasets for target detection. The images in the public dataset PASCAL VOC [15] and COCO [14] are collected in natural conditions with sufficient lighting, and the image quality is good. However, due to the limitation of the acquisition environment and other reasons, defective images of electronic components will be under-exposed or over-exposed during acquisition, which will introduce a considerable amount of noise components, resulting in low image quality. Low-quality images will affect the ability of target detection network to learn features to a certain extent, thereby affecting the effects of target detection. Therefore, after image preprocessing on training data set to improve the quality of image, it’s beneficial to enrich information of extracted features, thus improving defect detection in a certain part.

In this paper, in order to further improve the effect of electronic component surface defect detection, image pre-processing operation is required for the input image. If ordinary image noise reduction and restoration methods are used, it is very easy to mistake small target defects and detailed texture information as noise and remove them, and lose part of the feature information. Therefore, Real-ESRGAN [47] super-resolution processing is added to this network, which is a modeling process that utilizes higher-order degradations to simulate complex real-world degradations, while also takes into account ringing and overshoot that are common during synthesis artifacts and incorporate a U-Net discriminator with spectral normalization to improve the discriminator performance and stabilize training dynamic super-resolution methods. Based on this method, we have enlarged images in the original training set from 1392 × 1040 pixels to 5568 × 4160 pixels, which greatly improves the clarity of the images, and has richer texture information, feature information of significant regional, and boundary feature information.

#### 3.5.2 Data enhancement

The characteristics of the image of electronic components are also very different from those of public data sets. Whether it is the 20 categories of PASCAL VOC [14] or the 84 categories of the COCO [15] dataset, they are common items in daily life, and it is very convenient to collect, so the dataset is rich, the sample size is large, and the distribution of each category is uniform. The defects of electronic components are caused by the irregular operation of workers or equipment in actual industrial scenarios, and it is difficult to obtain defective samples, resulting in the characteristics of many positive samples and few negative samples, which has a significant impact on the detection task, particularly the classification task. And resulting in the overfitting of the training samples and the poor performance of the trained network weights in the testing phase. Therefore, the application of data enhancement in panel defect detection is particularly important. The effectiveness of data enhancement lies in expanding the data set, making the model more robust to images obtained in different environments. In order to handle geometric distortion, random scales, cropping, translation, shearing and rotation were added. Mixup and mosaic operations were combined for data enhancement at the same time. The Mosaic operation is an improved version of Cutmix, which greatly enriches the background of the detected object by stitching four images. Additionally, batch normalization computes activation statistics for four different images on each layer.

## 4. Experiments

In this section, we compare our proposed method with the current state-of-the-art target detection algorithms, including two-stage and one-stage target detection algorithms, using the labeled electronic panel defect dataset, in addition to effective ablation experiments, the details of which are described in detail in this section.

### 4.1 Data set

Our dataset was collected at an industrial site, and a total of 6155 electronic panel images were photographed and collected by vertical angle, and labeled according to the PASCAL VOC dataset format using labelImg target inspection dataset labeling software. In total, the 6155 images contain 370 defect-free images and 5785 defective images, with a total of 64 defect categories, where the training set, validation set, and test set are divided according to the ratio of 8:1:1, and the training set, validation set, and test set all contain defect images of each of the 64 defect categories.

### 4.2 Comparative experiment

**Fig 8:**
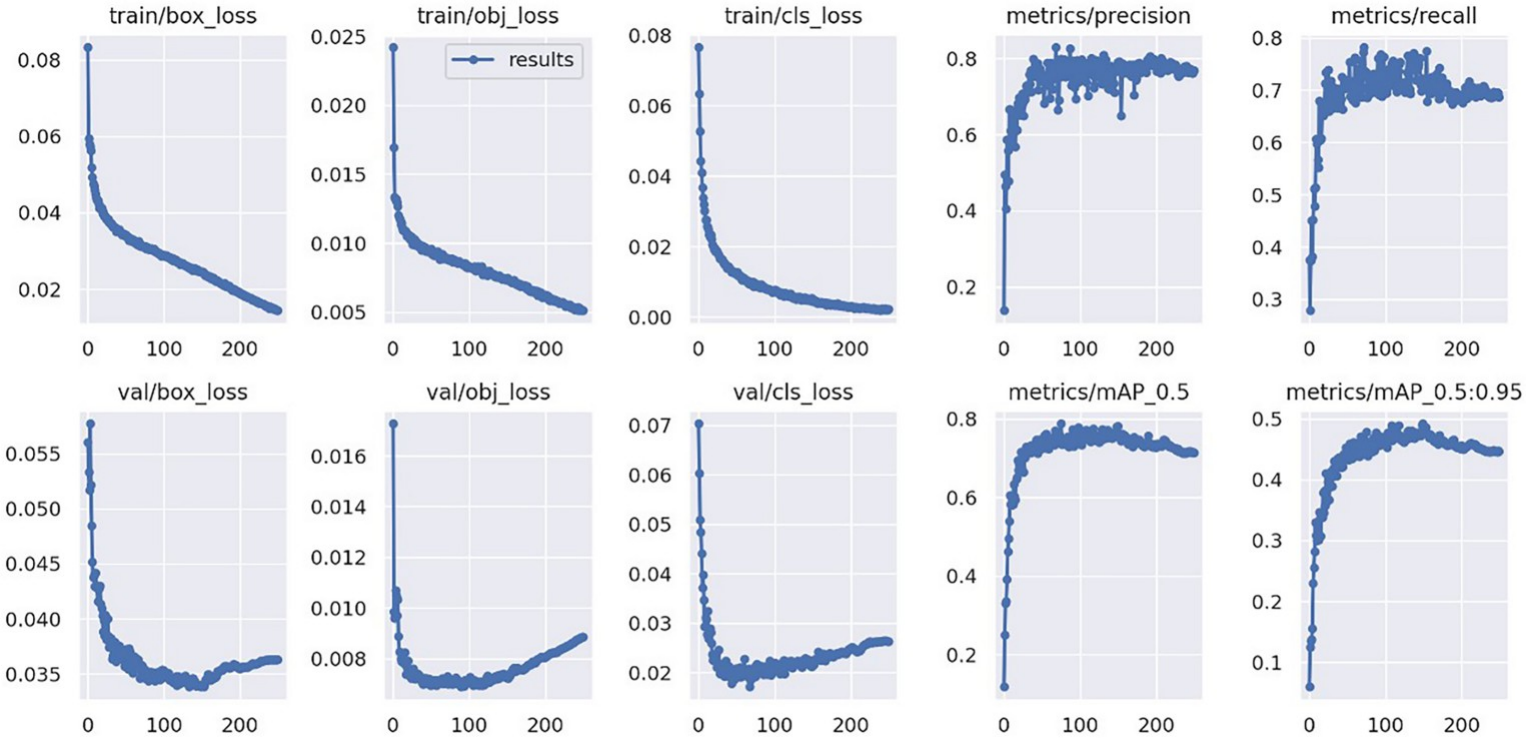
Training results of our designed network.

**Fig 9:**
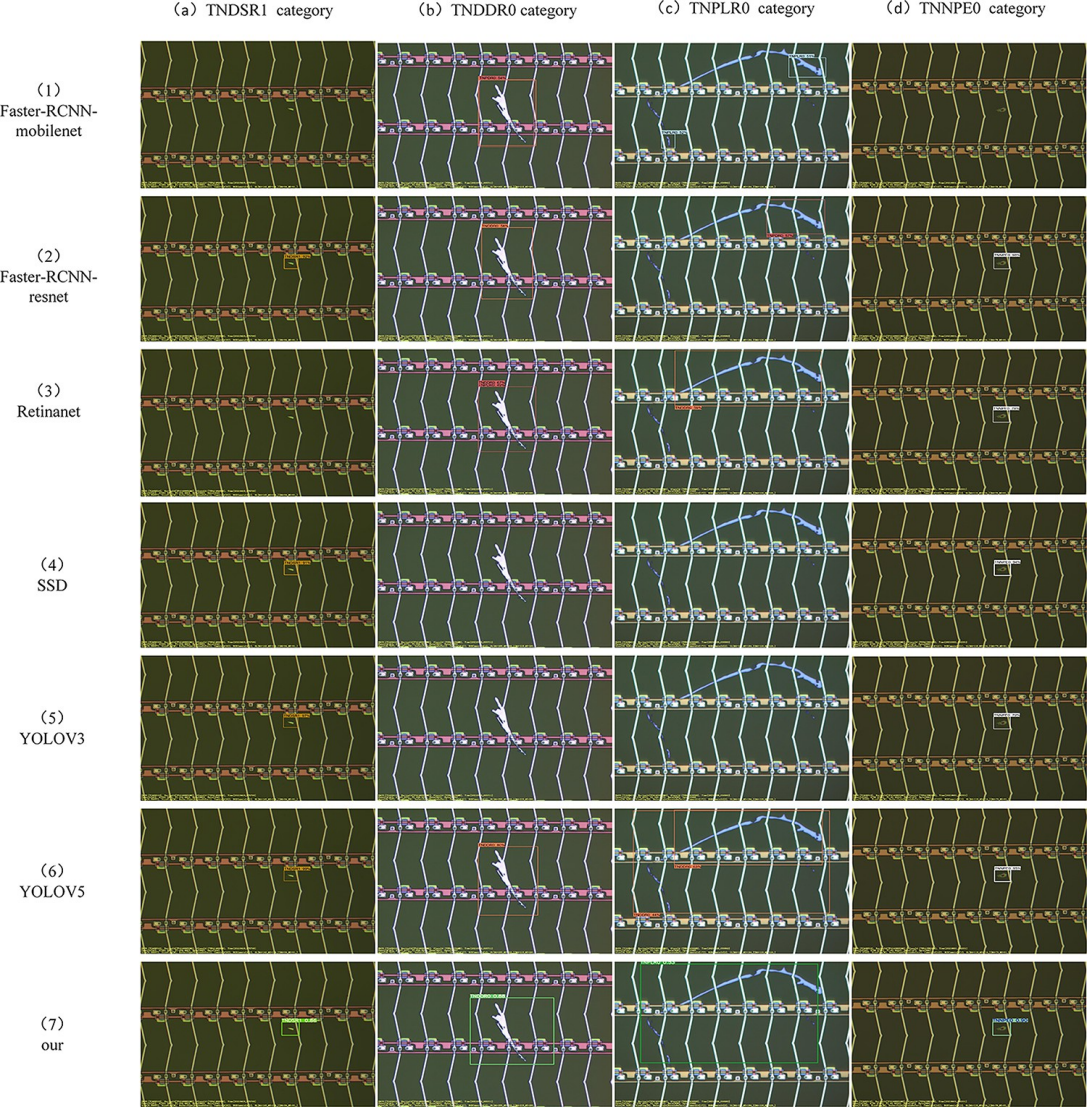
Test results of each trained network on the four categories of TNDSR1, TNDDR0, TNPLR0, TNNPE0.

In order to prove the effectiveness of our proposed network, we conducted experiments on our network with state-of-the-art object detection algorithms on the same dataset, including Faster-RCNN, Retinanet, SSD, YOLOV3, YOLOV5, and YOLOX. The network is trained on a PC with Nvidia GeForce RTX 2060 GPU. Our algorithmic network framework was selected from pytorch version 1.9.1, and we trained each group of experiments the same number of times, all with 200 epochs and a batch-size of 4. The size of the input images was uniformly cropped to 640*640, and the same image preprocessing and data enhancement operations were used. The training results of our designed network are shown in Fig 8, and the comparison with the training results of each network is shown in Table 1. In addition, we also randomly selected several representative small target defects and irregular defects from the test set of uninvolved network training to test each trained network, and the test results are shown in Fig 9.

**Table 1:** Comparison of the proposed method with Faster- RCNN, Retinanet, SSD, YOLOV3, YOLOV5, and YOLOX on the same dataset for each metric.

method	map_0.5	map_0.5:0.95	recall
Faster-RCNN (resnet50)	0.3828	0.2167	0.3752
Faster- RCNN (mobile-net)	0.3745	0.2116	0.3383
Retinanet	0.448	0.263	0.539
SSD	0.3095	0.1843	0.3143
YOLOV3	0.3684	0.22881	0.35226
YOLOV5	0.64751	0.39064	0.68898
YOLOX	0.6387	0.391107	0.67236
Ours	0.78257	0.49202	0.77509

From the above experimental results, it can be seen that the algorithm proposed in this paper outperforms the current state-of-the-art target detection algorithms in both the map index and the recall index, specifically, it improves 47.307 percentage points over SSD in the map_0.5 index, and 13.506 and 14.387 percentage points over the best YOLOV5 and YOLOX, which indicates the good applicability of the method for surface defect detection of electronic panels. In the test results, for the small target defects TNDSR1 and TNNPE0, Faster-RCNN (mobilenet) showed a missed detection on both TNDSR1 and TNNPE0, while Retinanet showed a missed detection on TNDSR1. For irregular defect TNDDR0, Faster-RCNN (mobilenet) and Retinanet showed false detection and duplicate detection on TNDDR0 category, while SSD and YOLOV3 did not detect TNDDR0 category defects. On irregular defects TNPLR0, Faster-RCNN (resnet) and Retinanet showed false detection on TNPLR0 category defects, SSD and YOLOV3 showed missed detection, and YOLOV5 showed repeated detection and inaccurate localization. In the above four categories, each network showed different degrees of false detection, missed detection or mislocation and repeated detection, while the network we designed accurately predicted the category and location of defects in all four defect categories with 86%, 88%, 53% and 90% confidence levels, respectively. In particular, the average test speed on the same test images is shown in Table 1, where our method achieves an average frame rate of 28 fps, which is only 2 fps and 4 fps lower than YOLOV5 and YOLOX, respectively. This shows that our method can still maintain a high speed while achieving a higher speed than other methods. The reason for this is that we have adopted a method that better fits the electronic panel surface defect detection scenario and has better interpretation, of which two points need to be emphasized. First of all, the surface defects of electronic panels show the characteristic of irregular, and the feature extraction modules of the networks in the comparison experiments are made by standard convolution stacking, which leads to the inability of accurate feature extraction for irregular defects, resulting in more false detection and missed detection. The T-deformable convolution can better adapt to the feature extraction of irregular defects by adaptive learning of the offset, which greatly reduces the probability of missed and false detection of electronic panel surface defects, thus improving the accuracy of detection at the overall level.

Second, most of the surface defects of electronic panels also have the characteristics of small targets. In the comparison experiments, although Faster-RCNN, Retinanet, and SSD use various feature pyramid structures to enrich feature information in the feature extraction stage, their single detection head structures are still not effective for better detection of small target defects. For YOLOV3, YOLOV5, and YOLOX, although these networks use a multi-detection head structure, the features captured at the detection end can no longer form an accurate mapping relationship with specific defect targets or background features due to the deepening of the network structure. Our designed double T-head structure, however, not only improves the detection effect on small target defects to a certain extent, but also improves the detection accuracy of generic defect targets by assigning different detection heads to targets of different scales, while combining the SE mechanism to form an accurate mapping of the corresponding defect targets or background features.

**Fig 10:**
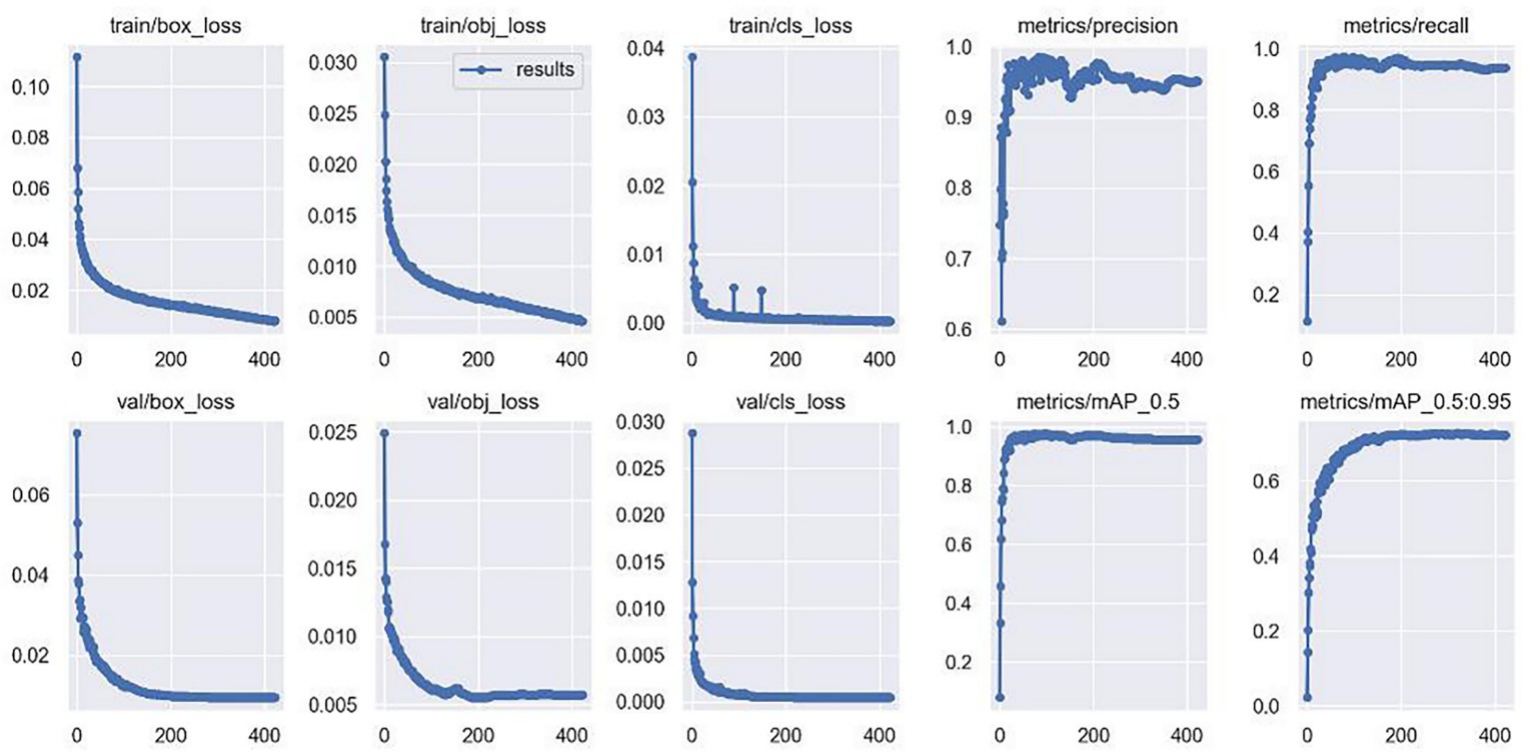
Training results of our network on the MSD dataset.

In addition, to demonstrate the generalization performance of the model, we selected the PCB defect dataset MSD [48] provided by the Intelligent Robot Development Laboratory of Peking University to conduct experiments on the generalization performance of the model. It mainly contains missing_hole, mouse_bite, open_circuit, short, spur, and spurious_copper 6 types of common PCB surface defects. The original dataset contained a total of 693 PCB defects, which we enhanced offline using rotation and scaling, and the dataset was expanded to 6136 images. Experiments were conducted to compare our network with each network. The training results of our network are shown in Fig 10. The comparison of the metrics of each network is shown in Table 2.

**Table 2:** Comparison experiments between the proposed method and each comparison method on the MSD data set.

method	map_0.5	map_0.5:0.95	recall
Faster-rcnn (resnet50)	0.7256	0.4115	0.6355
Faster-rcnn (mobile-net)	0.6114	0.3098	0.8895
retinanet	0.818	0.367	0.496
SSD	0.776	0.386	0.7713
YOLOv3	0.782	0.4074	0.7319
YOLOV5	0.9592	0.54642	0.9334
YOLOX	0.9503	0.6796	0.9684
Ours	0.97855	0.72801	0.9762

From the data in Table 2, it is clear that our proposed method has good generalization performance. In the experiments on the MSD dataset, the map_0.5 metric reaches 0.97855, the map_0.5:0.95 reaches 0.72801, and the recall metric reaches 0.9762, and the results are better than the other seven methods on these three metrics. There are two main reasons for this. First, the MSD dataset has only 6 categories, which makes the detection of various networks much less difficult compared to the original 64 categories of defects. But more importantly, the modules such as T-deformable convolution and double T-head designed by our network fully consider the feature extraction problem of various irregular targets and the detection problem of small targets. It can be seen that the map_0.5 index of the proposed method is 0.16055 higher than that of the retinanet, while the map_0.5:0.95 index is 0.36101 higher than that of the retinanet. this change has the same trend on the comparison of the other six methods with the proposed method, proving that the proposed method not only has good detection effect but also has good generalization performance. Next, we will conduct a series of ablation experiments to demonstrate the effectiveness of the designed T-deformable convolution and double T-head structure.

### 4.3 Ablation experiment

In order to verify the effectiveness of the proposed method, a series of ablation experiments are conducted on the basis of the proposed method, keeping both the hardware equipment and the algorithmic framework unchanged, and performing the same image preprocessing and image enhancement operations on all networks. The purpose of the ablation experiments is to verify the effectiveness of our designed T-deformable convolution and Double T-head, including replacing the T-deformable convolution with DCN, standard convolution, and using only Head1 detection head at the detection side. Table 3 shows the effects of each method on map metrics and recall metrics when performing ablation experiments.

**Table 3:** Map metrics and recall metrics for each network in the ablation experiment.

method	map_0.5	map_0.5:0.95	recall
Ours	0.78257	0.49202	0.77509
Replace T-deformable convolution with DCN	↓0.5	↓0.8	↓2.886
Replace T-deformable with Standard convolution	↓3.1	↓2.2	↓5.4505
Without Double T-head	↓0.35	↓0.873	↓1.362
Replace SE block with convolution block	↓0.14	↓0.362	↓0.4

The results in Table 3 show that when replacing our designed T-deformable convolution with DCN, its map_0.5 metric decreases by 0.5 percentage points, its map_0.5:0.95 metric decreases by 0.8 percentage points, and its recall metric decreases by 2.886 percentage points. This is mainly because DCN, compared to the T-deformable convolution we designed, has insufficient learning of offsets in the process of feature extraction, which tends to confuse the defective target features or background feature information, resulting in the inability to fully extract the feature information of irregular defects, thus causing a large number of false detections and missed detections on such targets, which in turn affects the overall detection accuracy. After replacing our designed T-deformable convolution with the standard convolution, its map_0.5 metric decreases by 3.1 percentage points, its map_0.5:0.95 metric decreases by 2.2 percentage points, and its recall metric decreases by 5.4505 percentage points. This is because the standard convolution is further weakened in its ability to extract features from irregular defects and more frequent false and missed detections occur compared to DCN and the T-deformable convolution we have designed. After removing head2 and keeping only head1, its map_0.5 metric decreased by 0.35 percentage points, its map_0.5:0.95 metric decreased by 0.873 percentage points, and its recall metric decreased by 1.362 percentage points. This is mainly because the additional design of Head2, after upsampling operation, increases the perceptual field and is able to map small-scale defects that cannot be mapped by head1, which in turn sends more feature information of small target defects to head2, enabling it to detect small target defects that cannot be detected by head1, improving the detection accuracy of small target defects. After replacing the SE block in Double T-head with the solution block, map_0.5, map_0.5: 0.95, and recall decreased by 0.14, 0.362, and 0.4, respectively. Because the addition of the SE mechanism can more effectively suppress the feature channels that are irrelevant to the target to be detected, enhance the weights of the target-related feature channels, improve the semantic information of the feature map, and thus improve the detection accuracy of the defective target. The results of the above ablation experiments prove that the proposed methods are all effective and can be better applied to scenarios where the defects of electronic panels are irregular and small targets.

### 5. Conclusion

In this paper, for the first time, we propose some targeted solutions to a series of problems that need to be solved in the detection of surface defects in electronic panels, including irregularity of defects and small targets, and these methods are highly innovative. Specifically, it includes the T-deformable convolution module designed for solving irregular defect problems and the Double T-head designed for solving small target problems. Through extensive comparison experiments and ablation experiments, it is shown that the proposed method is effective in scenarios with small targets and irregular defects in electronic panels, and it shows better detection accuracy than even the current state-of-the-art target detection methods while maintaining a faster detection speed, and also has a lower incidence of false and missed detections during the test. Also, we have confirmed the good generalization performance of the proposed method in the comparison experiments. We hope that this paper can bring some inspiration to the research work of defect detection in industry or academia. Next, we will conduct further research on unsupervised defect detection and computerized system of target detection algorithm, expecting to further improve the efficiency of defect detection and make some contribution to the industry.

## References

[1] LECUN Y, BENGIO Y, HINTON G. Deep learning[J]. Nature, 2015, 521(7553):436–444. 26017442 10.1038/nature14539

[2] Girshick R, Donahue J, Darrell T, et al. Rich feature hierarchies for accurate object detection and semantic segmentation[C]//Proceedings of the IEEE conference on computer vision and pattern recognition. 2014: 580–587.

[3] Girshick R. Fast r-cnn[C]//Proceedings of the IEEE international conference on computer vision. 2015: 1440–1448.

[4] Ren S, He K, Girshick R, et al. Faster r-cnn: Towards real-time object detection with region proposal networks[J]. Advances in neural information processing systems, 2015, 28: 91–99. doi: 10.48550/arXiv.1506.01497

[5] He K, Gkioxari G, Dollár P, et al. Mask r-cnn[C]//Proceedings of the IEEE international conference on computer vision. 2017: 2961–2969.

[6] Redmon J, Divvala S, Girshick R, et al. You only look once: Unified, real-time object detection[C]//Proceedings of the IEEE conference on computer vision and pattern recognition. 2016: 779–788.

[7] Redmon J, Farhadi A. YOLO9000: better, faster, stronger[C]//Proceedings of the IEEE conference on computer vision and pattern recognition. 2017: 7263–7271.

[8] Redmon J, Farhadi A. Yolov3: An incremental improvement[J]. arXiv preprint arXiv:1804.02767, 2018.

[9] Bochkovskiy A, Wang C Y, Liao H Y M. Yolov4: Optimal speed and accuracy of object detection[J]. arXiv preprint arXiv:2004.10934, 2020. doi: 10.48550/arXiv.2004.10934

[10] Xie E, Ding J, Wang W, et al. Detco: Unsupervised contrastive learning for object detection[C]//Proceedings of the IEEE/CVF International Conference on Computer Vision. 2021: 8392–8401.

[11] He K, Fan H, Wu Y, et al. Momentum contrast for unsupervised visual representation learning[C]//Proceedings of the IEEE/CVF conference on computer vision and pattern recognition. 2020: 9729–9738.

[12] Jin S Y, RoyChowdhury A, Jiang H, et al. Unsupervised hard example mining from videos for improved object detection[C]//Proceedings of the European Conference on Computer Vision (ECCV). 2018: 307–324.

[13] Liu Y C, Ma C Y, He Z, et al. Unbiased teacher for semi-supervised object detection[J]. arXiv preprint arXiv:2102.09480, 2021.

[14] Lin Tsung-Yi, Maire Michael, Belongie Serge, Hays James, Perona Pietro, Ramanan Deva, Dollar Piotr, and Lawrence C´ Zitnicket al. Microsoft coco: Common objects in context. In European conference on computer vision, pages 740–755. Springer, 2014.

[15] Everingham Mark, Luc Van Gool, Williams Christopher K. I., Winn John M., and Zisserman Andrew. The pascal visual object classes (VOC) challenge. Int. J. Comput. Vis., 88(2):303–338, 2010.

[16] Mei S, Wang Y, Wen G. Automatic fabric defect detection with a multi-scale convolutional denoising autoencoder network model[J]. Sensors, 2018, 18(4): 1064. 29614813 10.3390/s18041064 PMC5948749

[17] Zhao Z, Li B, Dong R, et al. A surface defect detection method based on positive samples[C]//Pacific Rim International Conference on Artificial Intelligence. Springer, Cham, 2018: 473–481.

[18] Ling Z, Zhang A, Ma D, et al. Deep Siamese Semantic Segmentation Network for PCB Welding Defect Detection[J]. IEEE Transactions on Instrumentation and Measurement, 2022, 71: 1–11.

[19] Han H, Gao C, Zhao Y, et al. Polycrystalline silicon wafer defect segmentation based on deep convolutional neural networks[J]. Pattern Recognition Letters, 2020, 130: 234–241.

[20] Hu J, Shen L, Sun G. Squeeze-and-excitation networks[C]//Proceedings of the IEEE conference on computer vision and pattern recognition. 2018: 7132–7141.

[21] He Y, Song K, Meng Q, et al. An end-to-end steel surface defect detection approach via fusing multiple hierarchical features[J]. IEEE Transactions on Instrumentation and Measurement, 2019, 69(4): 1493–1504.

[22] Wang T, Chen Y, Qiao M, et al. A fast and robust convolutional neural network-based defect detection model in product quality control[J]. The International Journal of Advanced Manufacturing Technology, 2018, 94(9): 3465–3471.

[23] Qiu L, Wu X, Yu Z. A high-efficiency fully convolutional networks for pixel-wise surface defect detection[J]. IEEE Access, 2019, 7: 15884–15893.

[24] Chen Y, Ding Y, Zhao F, et al. Surface Defect Detection Methods for Industrial Products: A Review[J]. Applied Sciences, 2021, 11(16): 7657.

[25] Hu B, Wang J. Detection of PCB surface defects with improved faster-RCNN and feature pyramid network[J]. Ieee Access, 2020, 8: 108335–108345.

[26] Mei S, Yang H, Yin Z. An unsupervised-learning-based approach for automated defect inspection on textured surfaces[J]. IEEE Transactions on Instrumentation and Measurement, 2018, 67(6): 1266–1277.

[27] Zhao Z, Li B, Dong R, et al. A surface defect detection method based on positive samples[C]//Pacific Rim International Conference on Artificial Intelligence. Springer, Cham, 2018: 473–481.

[28] Di H, Ke X, Peng Z, et al. Surface defect classification of steels with a new semi-supervised learning method[J]. Optics and Lasers in Engineering, 2019, 117: 40–48.

[29] Mujeeb A, Dai W, Erdt M, et al. Unsupervised surface defect detection using deep autoencoders and data augmentation[C]//2018 International Conference on Cyberworlds (CW). IEEE, 2018: 391–398.

[30] Hu G, Huang J, Wang Q, et al. Unsupervised fabric defect detection based on a deep convolutional generative adversarial network[J]. Textile Research Journal, 2020, 90(3–4): 247–270.

[31] Zhao Z, Li B, Dong R, et al. A surface defect detection method based on positive samples[C]//Pacific Rim International Conference on Artificial Intelligence. Springer, Cham, 2018: 473–481.

[32] Kim J, Ko J, Choi H, et al. Printed circuit board defect detection using deep learning via a skip-connected convolutional autoencoder[J]. Sensors, 2021, 21(15): 4968. 34372203 10.3390/s21154968 PMC8347834

[33] Gao Y, Gao L, Li X, et al. A semi-supervised convolutional neural network-based method for steel surface defect recognition[J]. Robotics and Computer-Integrated Manufacturing, 2020, 61: 101825.

[34] Hajizadeh S, Núnez A, Tax D M J. Semi-supervised rail defect detection from imbalanced image data[J]. IFAC-PapersOnLine, 2016, 49(3): 78–83.

[35] Gao Y, Gao L, Li X, et al. A semi-supervised convolutional neural network-based method for steel surface defect recognition[J]. Robotics and Computer-Integrated Manufacturing, 2020, 61: 101825.

[36] Long J, Shelhamer E, Darrell T. Fully convolutional networks for semantic segmentation[C]//Proceedings of the IEEE conference on computer vision and pattern recognition. 2015: 3431–3440.

[37] He K, Gkioxari G, Dollár P, et al. Mask r-cnn[C]//Proceedings of the IEEE international conference on computer vision. 2017: 2961–2969.

[38] Tabernik D, Šela S, Skvarč J, et al. Segmentation-based deep-learning approach for surface-defect detection[J]. Journal of Intelligent Manufacturing, 2020, 31(3): 759–776.

[39] Cao J, Yang G, Yang X. A pixel-level segmentation convolutional neural network based on deep feature fusion for surface defect detection[J]. IEEE Transactions on Instrumentation and Measurement, 2020, 70: 1–12.33776080

[40] Su-qin W, Qi R E N, Min S H I, et al. Product surface defect detection and segmentation based on anomaly detection[J]. Journal of Graphics, 2022, 43(3): 377.

[41] Li Y, Wang H, Dang L M, et al. A robust instance segmentation framework for underground sewer defect detection[J]. Measurement, 2022, 190: 110727.

[42] Dai J, Qi H, Xiong Y, et al. Deformable convolutional networks[C]//Proceedings of the IEEE international conference on computer vision. 2017: 764–773.

[43] Yu Jiahui, et al. "Unitbox: An advanced object detection network." Proceedings of the 24th ACM international conference on Multimedia. 2016.

[44] Rezatofighi Hamid, et al. "Generalized intersection over union: A metric and a loss for bounding box regression." Proceedings of the IEEE/CVF conference on computer vision and pattern recognition. 2019.

[45] Zheng Zhaohui, et al. "Distance-IoU loss: Faster and better learning for bounding box regression." Proceedings of the AAAI conference on artificial intelligence. Vol. 34. No. 07. 2020.

[46] Zheng Zhaohui, et al. "Enhancing geometric factors in model learning and inference for object detection and instance segmentation." IEEE Transactions on Cybernetics (2021). 10.1109/TCYB.2021.3095305 34437079

[47] Wang X, Xie L, Dong C, et al. Real-esrgan: Training real-world blind super-resolution with pure synthetic data[C]//Proceedings of the IEEE/CVF International Conference on Computer Vision. 2021: 1905–1914.

[48] Ding R, Dai L, Li G, et al. TDD‐net: a tiny defect detection network for printed circuit boards[J]. CAAI Transactions on Intelligence Technology, 2019, 4(2): 110–116.

